# Commentary: Increased circulating Th17 cells and altered CD4 T cell maturation and differentiation in active tuberculosis with type 2 diabetes: a pilot study

**DOI:** 10.3389/fimmu.2026.1750994

**Published:** 2026-01-23

**Authors:** Hygon Mutavhatsindi, Muki Shey

**Affiliations:** 1 Biomedical Research and Innovation Platform (BRIP), South African Medical Research Council, Tygerberg, South Africa; 2 Department of Medicine, Faculty of Health Sciences, University of Cape Town, Cape Town, South Africa; 3 Centre for Infectious Diseases Research in Africa (CIDRI-Africa), Faculty of Health Sciences, University of Cape Town, Cape Town, South Africa; 4 Institute of Infectious Disease and Molecular Medicine (IDM), Faculty of Health Sciences, University of Cape Town, Cape Town, South Africa

**Keywords:** CD4 T cell differentiation, immunometabolism, Th17 cells, tuberculosis, type 2 diabetes mellitus

## Introduction

The co-epidemic of tuberculosis (TB) and type 2 diabetes mellitus (T2DM) remains one of the major barriers to global TB eradication. T2DM triples the risk of developing active TB (1), complicates treatment, and is associated with delayed cure and increased mortality (2, 3). While epidemiological studies have long defined the clinical scope of this syndemic, the precise immunological mechanisms remain unclear. A recent pilot study by Ogongo and colleagues (4) provides compelling cellular-level insight into this issue, reporting increased frequencies of circulating Th17 cells and altered total CD4 T cell maturation and differentiation in active TB patients with T2DM. Their findings call for a shift in perspective, recognizing T2DM not merely as a metabolic comorbidity but as an active immunomodulatory condition with major implications for TB pathogenesis, diagnosis, and host-directed therapy (HDT).

## The compromised gatekeeper: T-cell dysfunction and LTBI progression

Protective immunity against *Mycobacterium tuberculosis (M. tuberculosis)* is essential and relies on a successful, sustained T helper 1 (Th1) response. The Th1 axis, driven by Interferon-γ (IFN-γ), is essential for macrophage activation and the formation of the granuloma, the structure responsible for containing latent infection.

The observation that T2DM leads to altered CD4 T cell maturation and differentiation is arguably the most impactful finding from a public health perspective, as it offers a molecular mechanism for the increased risk of latent TB infection (LTBI) progression to active TB. Sustained latency relies on a population of durable, highly functional Th1 memory T cells (5, 6). The diabetic microenvironment, characterized by chronic hyperglycaemia and systemic inflammation, fundamentally disrupts T-cell immunometabolism (7). T cells exposed to chronic hyperglycaemia become increasingly glycolysis-dependent, a metabolic state that favours dysfunctional differentiation trajectories and can promote exhaustion phenotypes (8). Studies have shown that antigen-specific T-cell responses are diminished in latent TB co-infected with T2DM (8–10).

If T2DM compromises the terminal differentiation and survival of these protective Th1 memory cells, the immune surveillance required to contain dormant bacilli within the granuloma is critically impaired. The failure to maintain a high-quality, long-lived Th1 population suggests a permissive immunological environment that facilitates *M. tuberculosis* reactivation, directly connecting the metabolic status of the host to the failure of adaptive immunity to maintain latency. It is important to note that the immunological profiles characterized in the pilot study by Ogongo and colleagues represent total circulating CD4+ T-cell phenotypes rather than *M. tuberculosis*-specific or lung-resident populations. While these peripheral markers provide valuable insights into systemic immune dysregulation in T2DM, they may not fully reflect the antigen-specific responses or the localized inflammatory landscape within the lung parenchyma.

## Immune deviation: Th17 and the failure to cure

In active TB, the prognosis in T2DM patients is consistently worse, characterized by delayed sputum conversion, higher rates of treatment failure, and increased tissue damage (2, 3). The observed pathological shift toward increased frequencies of circulating Th17 cells provides a systemic biological correlate for these adverse clinical outcomes (2, 3).

However, the role of Th17 cells in TB is complex and context-dependent. While early Th17-mediated responses are essential for mucosal immunity and the initial recruitment of protective neutrophils to the site of infection, the efficacy of this axis follows a “Goldilocks” principle where both deficiency and excess of IL-17 can lead to clinical detriment (11). In the context of active tuberculosis-diabetes mellitus (TB-DM), the observed Th17 expansion appears to be a detrimental immune deviation rather than an effective protective response.

Chronic, misdirected inflammation driven by IL-17 is strongly associated with the matrix metalloproteinase (MMP) cascade, including heightened MMP-9 and MMP-1 activity often observed in T2DM, which is the central driver of cavitary, destructive lung disease. We posit that the chronic metabolic inflammation inherent to T2DM serves as a pathological driver that shifts the Th17 response away from its protective homeostatic baseline toward the hyper-inflammatory, maladaptive phenotype reported by Ogongo and colleagues. The excessive tissue pathology seen in TB-DM co-infection, often leading to permanent lung damage and functional decline is thus explained not by a lack of inflammation, but by an inappropriate type of inflammation.

**Figure 1:**
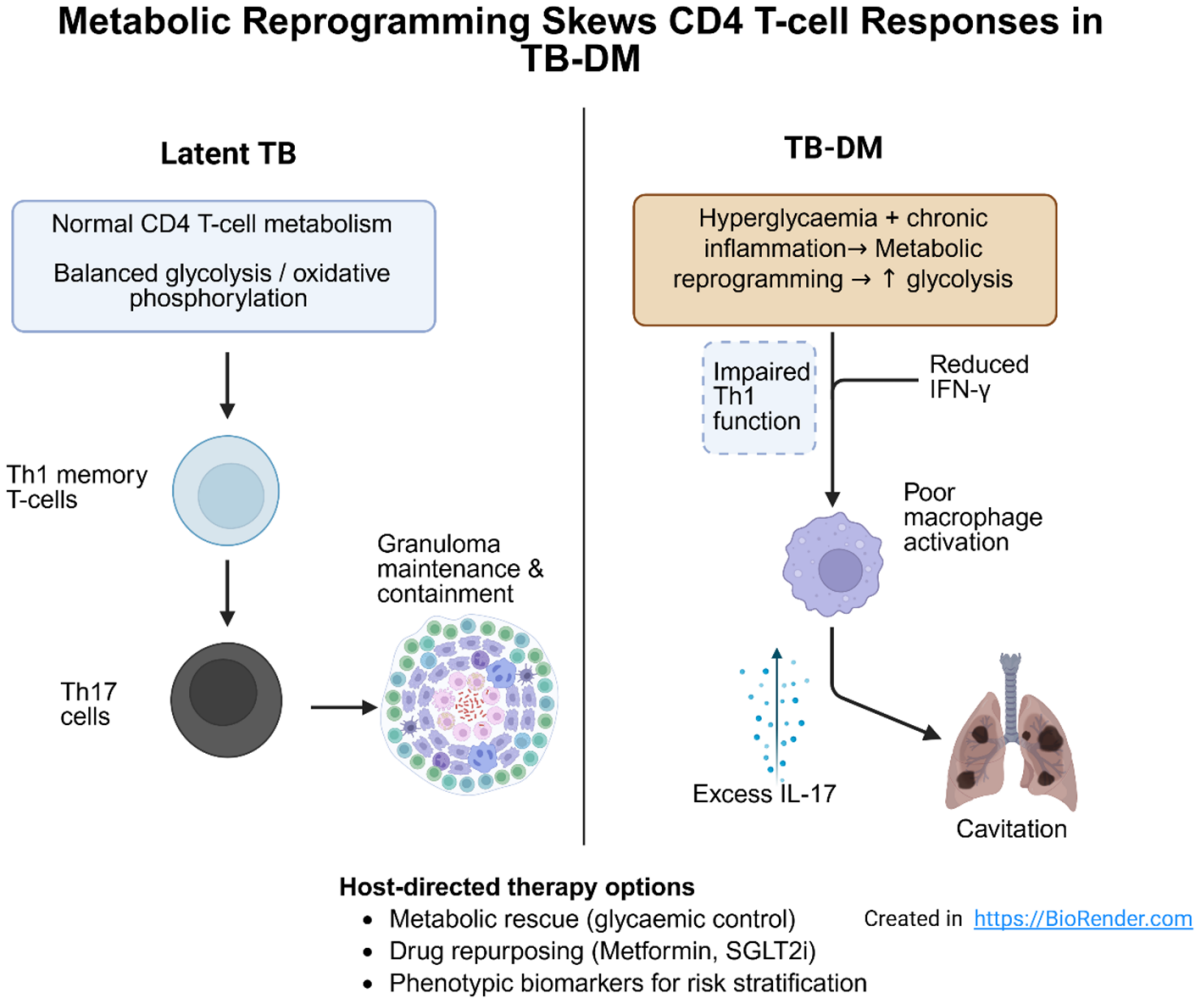
Metabolic reprogramming skews CD4 T-cell responses in TB-DM. High glucose and systemic inflammation in TB-DM patients reprogram CD4 T-cell metabolism, skewing the immune response from protective Th1 memory cells toward pathogenic Th17 cells. This shift contributes to impaired granuloma maintenance, tissue damage, and adverse clinical outcomes. Host-directed therapy strategies aim to restore Th1 function and reduce Th17-mediated pathology (15).

Furthermore, this deviation skews the T-cell differentiation landscape toward the Th17 lineage, often at the expense of the protective Th1 lineage. These findings suggest that T2DM may exert a dual detrimental effect, potentially attenuating the Th1-mediated protective response while amplifying Th17-driven inflammatory pathology. This overall imbalance between impaired Th1 immunity and exaggerated Th17 inflammation in TB-DM is summarized conceptually in **Figure 1**.

## Translating cellular findings into host-directed therapy

These findings transition T2DM in the context of TB from a passive comorbidity to an active immune-metabolic disorder that detrimentally programs the host immune response (3, 7). This perspective demands a strategic pivot in therapeutic design (12):

1. Metabolic rescue of T-cells

If hyperglycaemia and associated metabolic stress are driving T-cell dysfunction, then intensive, individualized glycaemic control must be viewed as an indispensable form of HDT for TB-DM patients (12). We must move beyond simply monitoring HbA1c to ensuring that acute metabolic fluctuations are minimized, thereby reducing the environmental cues that favour Th17 differentiation and T-cell exhaustion.

2. Drug repurposing

The immunomodulatory effects of established DM medications must be systematically investigated. Drugs like Metformin have known pleiotropic effects on T-cell metabolism, potentially promoting Th1 responses or dampening Th17 differentiation through pathways like AMP-activated protein kinase (AMPK) (13). Exploring the impact of newer DM therapies, such as Sodium-Glucose Co-Transporter (SGLT) 2 inhibitors, on T-cell differentiation and cytokine profiles in TB-DM models is also crucial (14).

3. Phenotypic biomarkers

The goal is moving beyond broad DM screening to immunological risk stratification. We propose that developing simple, robust phenotypic markers, for instance specific cell surface markers associated with circulating Th1 exhaustion or Th17 fate, could identify DM patients with LTBI who are at the highest, most imminent risk of progression, allowing for targeted preventative therapy.

## Conclusion

The pilot study on Th17 expansion and altered CD4 T cell differentiation provides important preliminary mechanistic insight, providing a much-needed molecular anchor for the devastating clinical reality of the TB-DM syndemic (4). T2DM does not merely coexist with TB; it reprograms the host adaptive immune system towards an ineffective total Th1 response and a destructive Th17-driven inflammatory state. Translational research must now urgently focus on metabolic and immunological rescue of the CD4 T cell population. By reversing these specific cellular defects, we can restore the immune system’s protective function, mitigate the risk of reactivation, and finally achieve durable cures in this challenging patient group. Ultimately, the pathways discussed herein represent theoretical targets that require rigorous validation in clinical settings to overcome the significant barriers of human patient variability and model fidelity.
